# Acute isolated acetabular fracture following a game of squash: a case report

**DOI:** 10.1186/1752-1947-1-156

**Published:** 2007-11-28

**Authors:** Neil D Patel, Ravi K Trehan

**Affiliations:** 1 Specialist Registrar, Trauma and Orthopaedics, Kettering General Hospital, Kettering, UK; 2 Specialist Registrar, Trauma and Orthopaedics, St George's Hospital, London, UK

## Abstract

Although hip injuries do not account a large amount of the Sports Physician's workload they can result in significant morbidity. We present a case where an acetabular fracture was sustained in a relatively young female while playing squash without any history of fall or injury but was treated successfully non-operatively. Such patients who present with acute hip pain must not be dismissed as simply having a soft tissue injury.

## Introduction

The number of hip joint injuries that present to the Sports Physician is relatively small, in comparison to other joints such as the knee ankle and shoulder, but their morbidity can be significant especially if the correct diagnosis is not made [1]. We describe a squash player who presented with acute hip pain during a game without any history of fall or injury, which turned out to be an isolated fracture of the acetabulum.

## Case presentation

A 47-year-old pre menopausal female presented to our Emergency Department with acute left hip pain while playing squash. The pain came on suddenly during a game after she lunged for a corner shot. The pain was so severe that she was unable to weight bear. She was an active club player for four years and was playing a club game when the incident occurred. She denied any previous hip injury or preceding hip and groin pain. Her level of physical activity had not altered recently and was otherwise fit and well.

**Figure 1:**
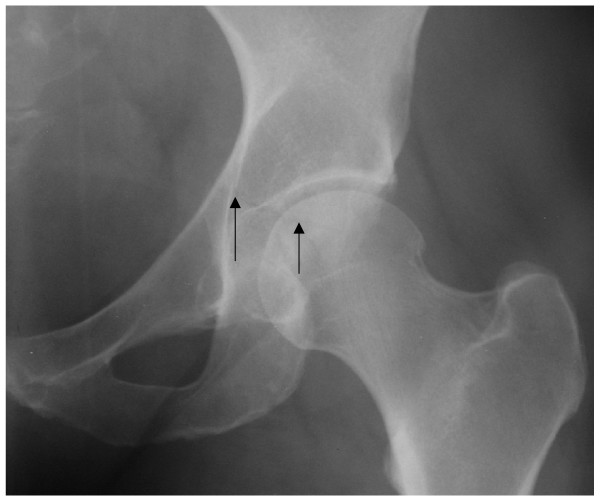
X ray of the left hip showing the suspicious fracture line extending across the acetabulum (arrows).

**Figure 2:**
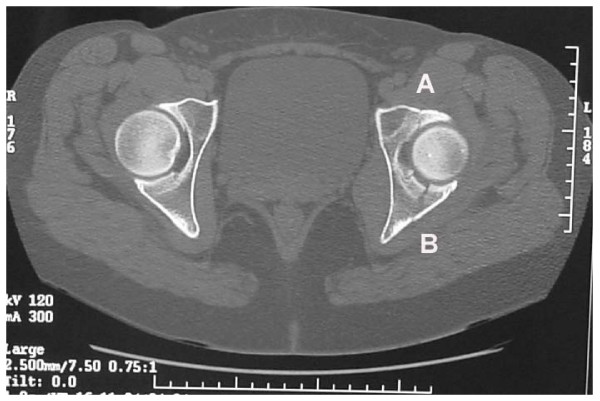
CT scan of the hip. Note the fracture line involving the anterior column (A) and the posterior column (B).

Examination revealed no deformity of the left leg. She complained bitterly of hip pain that was worse on passive movement. A plain radiograph revealed a suspicious line through the acetabulum (Fig 1). As the local unit had a policy of not performing Judet views a Computerised Tomography (CT) scan was then performed (Fig 2) and showed a minimally displaced fracture (<2 mm step off) of the acetabulum that had involved both columns and had extended into the dome as described by Olson and Matta [2]. A general examination and blood investigations revealed no evidence of infection, malignancy or metabolic disorders such as osteomalacia which may have contributed to the injury.

### Outcome

Management was conservative involving one week of bed rest to settle the acute pain sufficiently to allow non weight bearing mobilisation safely for further eight weeks. The patient was monitored closely for any displacement of the fracture [3] with repeat x rays at 2, 6 and 12 weeks. The patient was then allowed to mobilise fully through this hip as tolerated. Although she had regained pain free movements of the hip she hasn't, to date, returned to the squash court. A subsequent bone mineral density scan showed she had a T-score that was greater than -1 [4].

## Discussion

Squash is a moderate to high intensity sport that requires a certain level of fitness. When musculoskeletal injuries occur they commonly involve the lower limb and back[5]. As a weight-bearing joint, the hip is fundamental to the participation of sports such as squash. It is important in running and jumping as well as the twisting and turning movements needed for racquet sports. During normal weight bearing the hip joint is subjected to substantial forces. This can increase three to five times body weight during the movements described [6]. There are a wide variety of acute, sub acute and chronic injuries affecting the hip joint as well as the surrounding soft tissues. Establishing a correct diagnosis can, thus, be difficult [1]. Injuries to the hip joint include acetabular labral tears [7], which have been associated with sudden twisting or pivoting motions and may contribute to the progression of hip arthritis [8]. Presentation can be acute, but more commonly present insidiously with persisting or escalating symptoms. Similarly a stress fracture of the femoral neck can present with a gradual deterioration of symptoms [9] and if left untreated may progress to a displaced fracture with significant long-term morbidity. Stress fractures of the acetabulum are rare, but have been described in military endurance athletes and recruits [10]. The classification and treatment of fractures involving the acetabulum are well described with management based largely on the work done by Letournel [11]. Assessment of the continuity of the weight bearing dome of the acetabulum can be achieved by the measurement of the roof angles on plain radiographs [12] or by CT scan [2]. Patients are often involved in high-energy events such as road traffic accidents where other injuries frequently coexist. Those with metabolic or neoplasic conditions are more likely to sustain fractures from accidents where the transmission of energy through the hip joint is lower. To the author's knowledge acute isolated fracture of the acetabulum simply from playing squash has not been reported. This case suggests that the transmission of forces through the hip joint during a match is sufficient to cause acetabular fractures in certain individuals.

Bone mineral density is often quoted as a T score. A T score is the number of standard deviations below the mean of a fit premenopausal woman but does not take into account age, weight or ethnicity. The risk of sustaining a fracture increases by a factor of 2 to 3 per T unit [4]. This individual had a bone mineral density within normal parameters and a history which makes the probability of a pre-existing stress fracture small. Despite this, however, it would seem that the transmission of forces through the hip joint during a match is sufficient to cause acetabular fractures in certain individuals.

## Conclusion

When dealing with acute hip pain, following high intensity and high impact activities, a high index of suspicion must be given to the presence of a significant joint injury and not simply be dismissed as soft tissue pathology. Successful treatment of this type of fracture, as shown here, can be achieved non-operatively. However the long term prognosis, with regards to the development of hip pain and degenerative changes, is unknown. Physicians need to be aware of the risk of serious hip injury to those individuals presenting with sports related hip pain who undertake high impact activities later in their lives.

## Competing interests

The author(s) declare that they have no competing interests.

## Authors' contributions

NP was involved in the case directly, performed the literature search and drafted part of the manuscript.

RT was involved in the literature review and helped draft the manuscript.

All authors read and approved the final manuscript.

## Consent

The patient's informed written consent has been obtained for publication of this manuscript.
